# Severity matters: Using network analysis to explain low and high levels of persecutory beliefs

**DOI:** 10.1016/j.scog.2026.100435

**Published:** 2026-03-25

**Authors:** S. Denecke, A. Bott, F. Strakeljahn, J. Kingston, T.M. Lincoln

**Affiliations:** a University of Hamburg, Clinical Psychology and Psychotherapy, Germany; b Royal Holloway University of London, Health and Wellbeing, United Kingdom

**Keywords:** Persecutory beliefs, Delusions, Aetiology, Network models

## Abstract

Delusions, including persecutory beliefs, are theorised to arise from multifaceted interacting mechanisms. In our previous machine-learning study, 55 theory-derived predictors appeared to better account for lower levels of persecutory beliefs, whereas higher severity scores were underestimated. This raises the question of whether aetiological predictors differ in their relevance across the severity continuum. In this secondary analysis of data from a large, stratified online sample (*N* = 336), we applied network analyses using the ten strongest predictors from the prior model to compare their associations with low versus high levels of persecutory beliefs. Consistent with earlier findings, predictors accounted for more variance in subclinical than in clinically high persecutory beliefs. Association patterns also differed across levels of severity: In the low-severity network, persecutory beliefs were directly linked to aberrant salience and ostracism, whereas in the high-severity network, persecutory beliefs were most strongly linked to negative beliefs about mistrust and threat anticipation. These observations indicate that some findings from subclinical samples may not generalise to clinical populations. The emergence of distinct symptom dynamics across the continuum underscores the need for more targeted investigation, particularly at the more severe end of the delusion continuum. Future longitudinal, temporally fine-grained network analyses will be crucial for clarifying how mechanisms shift as persecutory beliefs intensify.

## 1 Introduction

Over the last decades, cognitive behavioural therapy has demonstrated small-to-medium effects for the treatment of psychotic disorders, which are not maintained over more extended periods of time (Berendsen et al., 2024; Mehl et al., 2015). Given that over 50 theoretical models have been proposed to explain the mechanisms underlying the development of delusions, including persecutory delusions (Denecke et al., 2024), the limited progress in treating them appears puzzling. As outlined in a recent review, the proposed theoretical models adopt diverse perspectives and emphasise different mechanisms, yet converge on a core principle: that delusions arise as explanations for anomalous experiences (Denecke et al., 2024). Across models, a wide range of factors are thought to contribute to delusion formation, including cognitive factors, social influences, Bayesian inference processes, neurobiological disruptions, and mechanisms of associative learning. Collectively, these diverse predictors reflect an expanding body of research that suggests progress has been made in elucidating the mechanisms underlying delusion formation.

However, in a recent comprehensive machine-learning study, we found that 55 predictors derived from these theoretical models accounted for only 31% of the variance across the continuum of persecutory beliefs (Denecke et al., 2024). An exploratory binary classification model that predicted whether individuals scored above or below the clinical cutoff (≥ 11) on the Revised Green et al. Paranoid Thoughts Scale (R-GPTS; Freeman et al., 2021) achieved acceptable accuracy (68%). However, visual inspection of the predicted individual scores indicated better performance at lower compared to higher levels of persecutory beliefs. This raises the question of whether predictors are differentially associated with persecutory beliefs at lower versus higher severity levels, which would question the generalisability of theoretical models of delusions across the continuum, as well as the usefulness of subclinical samples for studying delusion development.

To further enhance the effectiveness of interventions for delusions, we require a more nuanced understanding of the mechanisms underlying delusion formation, particularly at the more severe end of the delusion continuum. Thus, it is necessary to determine how aetiological predictors relate to persecutory beliefs across different levels of severity. Based on the idea that mental disorders can be modelled as dynamic networks of interconnected symptoms, representing systems rather than entities, network analyses provide a valuable tool for unravelling such differences (Borsboom et al., 2019; Borsboom and Cramer, 2013; Kendler et al., 2011). Applied to psychosis and schizophrenia, this perspective has been used to examine dynamic interactions among symptoms, cognition, and contextual factors, suggesting that feedback processes may contribute to symptom maintenance (Badal et al., 2021; Badal et al., 2023; Berisha et al., 2025; Hardy et al., 2021). Against this background, this study follows up on Denecke and colleagues (2025) by using network analyses to (Q1) examine the extent to which the best-discriminating predictors explain variance in low and high levels of persecutory beliefs and (Q2) assess whether the associations differ in number and magnitude across the severity levels of persecutory beliefs. Estimating networks separately for low and high levels of persecutory beliefs allows the pattern of associations among predictors to vary between severity strata. This enables us to empirically examine whether the predictors proposed in theoretical models operate similarly across the continuum, thereby providing a deeper understanding of the dynamic processes underlying differences in predictability.

## 2 Methods

### 2.1 Participants and procedure

As part of a comprehensive study (Denecke et al., 2025), a gender-balanced pool of adults (aged 18 years or older) fluent in English and residing in the UK was recruited via Prolific (www.prolific.com). Participants provided informed consent before completing an online screening that assessed their age, sex assigned at birth, self-identified gender, and persecutory beliefs (Revised Green Paranoid Thoughts Scale [R-GPTS], Persecution subscale; Freeman et al., 2021). Based on the R-GPTS Persecution scores, participants were quota-sampled into the following subgroups: average (≤5), elevated (6–10), moderate (11–17), and severe/very severe (≥18). Eligible participants received invitations to the main survey within 24 h, with a median delay of 9.29 h (median absolute deviation = 11.71) between screening and participation in the main survey. Complete data were obtained from 336 participants (*n* = 84 per quota), corresponding to a power analysis for multiple regression (f^2^ = 0.15, ß = 0.95; G*Power 3.1.9.7; Faul et al., 2009). For the present study, the cutoff proposed by Freeman and colleagues (2021) to distinguish between clinical and non-clinical persecutory beliefs was used to divide participants into low (<11; *n* = 168) and high (≥11; n = 168) persecutory beliefs.

Both screening and main surveys were conducted via PsyToolkit (Stoet, 2010; Stoet, 2017), a validated platform for online experiments (Kim et al., 2019). To mitigate order effects, questionnaires, paradigms, and items were presented in a randomised order. The median durations were 1 min for the screening (*M* = 1.37, *SD* = 0.81) and 96 min for the main survey (*M* = 106.18, *SD* = 31.43), respectively. Participants received monetary compensation via Prolific (£0.12 for screening, £14 for the main study).

### 2.2 Predictors and materials

Participants completed the Persecution subscale of the R-GPTS (Freeman et al., 2021) to assess persecutory beliefs. This subscale comprises 10 items assessing persecutory beliefs over the past week (e.g., “I was sure someone wanted to hurt me”) and is rated on a Likert scale from 0 = *not at all* to 4 = *completely*. The R-GPTS is widely utilised in research on persecutory beliefs due to its strong psychometric properties (Statham et al., 2019). In our sample, the internal consistency for the Persecution subscale was excellent, with a Cronbach's alpha of α = 0.90.

As part of a larger study, we previously reported on a binary classification analysis that predicted which theory-derived predictors were most relevant for distinguishing low from high persecutory beliefs and could explain the observed difference in predictive accuracy across severity groups (Denecke et al., 2025). Specifically, in these previous analyses, random forests were used to predict whether individuals scored below (<11) or above (≥11) the clinical cutoff for persecutory beliefs (R-GPTS Persecution), using nested cross-validation to mitigate overfitting (Denecke et al., 2025). Further, each predictor's importance was evaluated using SHapley Additive exPlanations (SHAP; Lundberg and Lee, 2017). Based on these previous analyses, we utilised network analysis to examine whether differences in network structure account for differences in predictive accuracy across severities. Since the top ten most essential variables identified in our previous analysis (Denecke et al., 2025) accounted for the majority of the variance in our predictions, they were used to build the networks. These included aberrant salience, cognitive fusion, emotion regulation difficulties, hallucinations, negative beliefs about mistrust, ostracism, social anxiety, stress, stress reactivity, and threat anticipation (Denecke et al., 2025). All measures of the putative predicting variables are summarised in Table 1. Individual sum scores were used as variables for building the networks.

**Table 1:** Overview of the utilised measures of the predicting variables.

Variable	Instrument	N° items	Scale (anchors)	Internal consistency Cronbach's α	Example item
Aberrant Salience	Aberrant Salience Inventory (ASI; Cicero et al., 2010)	10	Yes (1)/ No (0)	0.82	“Do normally trivial observations sometimes take on an ominous significance?”
Cognitive Fusion	Cognitive Fusion Questionnaire-7 (CFQ-7; Gillanders et al., 2014)	7	7-point (1–7)	0.96	“I tend to get very entangled in my thoughts”
Emotion Regulation	Difficulties in Emotion Regulation Scale (DERS-16; Bjureberg et al., 2016)	16	5-point (1–5)	0.95	“When I am upset, I feel out of control”
Hallucinations	Launay-Slade Hallucination Scale- Modified II (LSHS-II; Larøi and Van Der Linden, 2005)	16	5-point (0–4)	0.91	“I have been troubled by hearing voices in my head”
Negative Beliefs about Mistrust	Beliefs about Paranoia Scale (BaPS) - negative subscale (Gumley et al., 2011)	6	4-point (1–4)	0.88	“Thoughts about suspiciousness worry me.”^a^
Ostracism	Ostracism Short Scale (OSS; Rudert et al., 2020)	4	7-point (1–7)	0.93	“Others ignored me”
Social Anxiety	Brief Fear of Negative Evaluation (BFNE-II; Carleton et al., 2007)	8	5-point (0–4)	0.96	“I am afraid that others will not approve of me”
Stress	Depression, Anxiety, and Stress Scale (DASS-21; Lovibond and Lovibond, 1995)	7	4-point (0–3)	0.88	“Over the past week, I found it hard to wind down”
Stress Reactivity	Arousal Predisposition Scale (APS; Coren, 1988)	12	5-point (1–5)	0.87	“I startle easily”
Threat Anticipation	Negative Events Scale (NES; Kaney et al., 1997; Reininghaus et al., 2019)	10	7-point (1–7)	0.92	“How likely is it that the following things will happen to you in the future? Someone complains about your work”

a The original items were rephrased from paranoia to suspiciousness, to include individuals with mild persecutory beliefs who might not identify their thoughts as paranoid.

### 2.3 Statistical analyses

After predictor selection based on the previous machine learning results reported in Denecke et al. (2025), we conducted network analyses to examine differences in associative networks between individuals scoring below and above a clinical cutoff, constructing independent networks for high and low levels of persecutory beliefs. In such networks, variables are represented by nodes, and associations between them by edges, with edge weights corresponding to undirected regularised partial correlations.

The networks were estimated using mixed graphical models in *R* (version 4.4.3), as implemented in the *mgm* package (Haslbeck and Waldorp, 2020). To minimise the risk of retaining false positives and to estimate sparse networks, this package implements the least absolute shrinkage and selection operator (LASSO), which shrinks weak or unstable correlations towards zero (Tibshirani, 1996). The strength of the LASSO penalty is selected via the Extended Bayesian Information Criterion (EBIC; Foygel and Drton, 2010), which is regulated by a modifiable tuning parameter *γ.* We calculated and compared the predictability (i.e., the extent to which connected nodes explain variance in a node, similar to *R*^*2*^) of edge weights for three values of *γ*, corresponding to a liberal, medium, and conservative penalty (i.e., 0, 0.25, and 0.5). Since predictability did not differ substantially across these values, we followed the recommendations of Foygel and Drton (2010) and selected a medium *γ* of 0.25 to balance retaining true associations with estimating a parsimonious network. All variables were *z*-transformed. The *qgraph* package (Epskamp et al., 2012) was used to visualise the networks.

The predictability (*R*^*2*^) of the persecutory beliefs nodes was compared to assess whether the networks differentially accounted for variance in low and high levels of persecutory beliefs (Q1). The edge weights of direct associations with persecutory beliefs were examined to assess whether these associations differ across levels of persecutory belief severity (Q2). Further, to evaluate and compare the extent to which persecutory beliefs are connected to other nodes within the two networks, we calculated three indices of centrality: 1) *strength* (i.e., the magnitude of all direct associations with other nodes), with higher values indicating a stronger connection to all other variables, 2) *betweenness* (i.e., the proportion of cases where the node lies along the shortest path between other nodes), with higher values indicating higher connectivity within the network, and 3) *closeness* (i.e., the magnitude of all indirect associations with other nodes), with higher values indicating a closer association with other nodes in the network. Since we were primarily interested in persecutory beliefs, we focused on comparing the centrality of the persecutory beliefs node across the two networks. Furthermore, we evaluated the networks' accuracy and stability using the recommended bootstrapping procedures (Epskamp et al., 2017). That is, we utilised the *estimateNetwork* and *bootnet* functions (Epskamp et al., 2017) to conduct bootstrapping (1000 bootstrapped samples) with 95% confidence intervals to assess the accuracy of the edge weights. Furthermore, we employed case-dropping bootstrapping (i.e., subsetting participants) to assess the stability of the centrality indices, using the recommended cutoff of 0.5 for the *CS-coefficients* (i.e., the maximum proportion of *n* that can be dropped while retaining a correlation >0.7 with the original centrality with 95% certainty).

## 3 Results

### 3.1 Sample characteristics

The sample comprised 336 participants, with a mean age of 39.5 years (SD = 12.9; Min = 18, Max = 76). Just over half identified as male (50.6%), 47.9% as female, 1.2% as non-binary and 0.3% selected “none apply”. In terms of education, the majority (56%) held a university degree, while 44% reported that their highest level of education was up to A-levels or equivalent. About a third of the sample reported a psychiatric diagnosis, with 0.6% reporting a diagnosis of a psychotic disorder and 32.7% reporting another mental health disorder. 19.6% of the sample reported receiving mental health treatment. The sample predominantly identified as white (87.2%), with smaller proportions identifying as Asian (5.4%), Mixed (3.9%), Black (1.8%), or another group (0.9%). Detailed characteristics by subgroup can be found in Supplement 1 (Table S1). The means and standard deviations of the predictor variables are presented in Table 2.

**Table 2:** Means and standard deviations of the predictor variables.

	Mean (SD)		
Variable	Total sample^1^	Low PB sample^2^	High PB sample^2^
Persecutory Beliefs (R-GPTS)	11.7 (8.6)	4.7 (3.7)	18.6 (6)
Aberrant Salience (ASI)	5.4 (3)	4.4 (2.8)	6.5 (2.7)
Cognitive Fusion (CFQ-7)	28.4 (10.5)	24.5 (10.5)	32.2 (9)
Emotion Regulation Difficulties (DERS-16)	40.9 (14.8)	35.6 (13.7)	46.1 (14)
Hallucinations (LSHS-II)	22.7 (15)	17.9 (13.4)	27.5 (15.1)
Negative Beliefs about Mistrust (BaPS - negative)	11.4 (4.3)	9.8 (3.9)	12.9 (4)
Ostracism (OSS)	12.1 (6.1)	10.2 (5.7)	14.0 (6)
Social Anxiety (BFNE-II)	17.1 (9.1)	14.4 (9.1)	19.8 (8.3)
Stress (DASS-21 - Stress)	15.2 (9.2)	12.0 (8.8)	18.5 (8.4)
Stress reactivity (APS)	35.6 (8.3)	32.9 (7.7)	38.2 (8)
Threat Anticipation (NES)	32.7 (12.4)	28.9 (11.2)	36.5 (12.4)
^1^N = 336, ^2^n = 168			

Note. PB = persecutory beliefs, R-GPTS = Revised Green et al. Paranoid Thoughts Scale, ASI = Aberrant Salience Inventory, CFQ-7 = Cognitive Fusion Questionnaire-7, DERS-16 = Difficulties in Emotion Regulation Scale 16, LSHS-II = Launey-Slade Hallucination Scale II, BaPS negative = Beliefs about Paranoia Scale - negative subscale, OSS = Ostracism Short Scale, BFNE-II = Brief Fear of Negative Evaluation Scale II, DASS-21- Stress = Depression Anxiety Stress Scales 21 - Stress subscale, APS = Arousal Predisposition Scale, NES = Negative Events Scale.

### 3.2 Network models and predictability

**Fig. 1:**
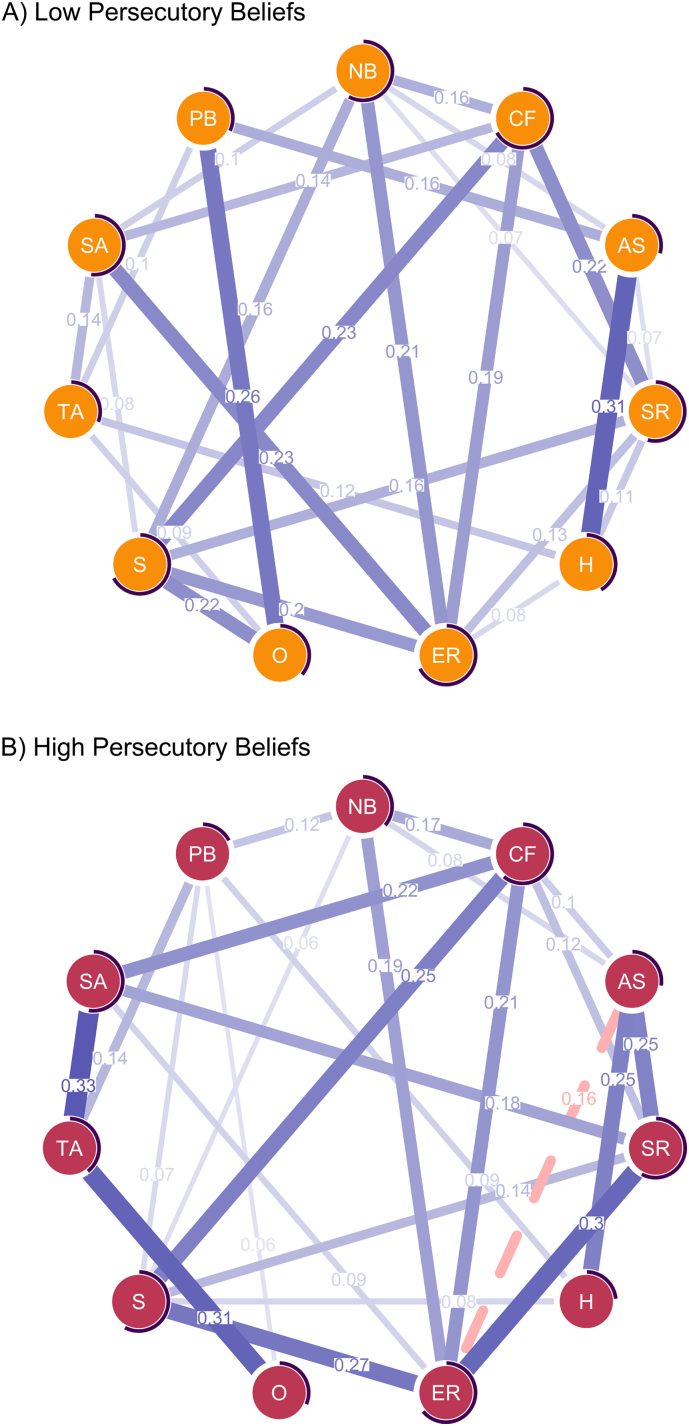
*Networks for the A) low and B) high persecutory belief samples.* *Note*. Positive associations are shown in blue (solid), and negative associations are shown in red (dashed). The width of an edge indicates the strength of the association between two variables (i.e., edge weight). The circle around each node represents the explained variance (*R*^*2*^). SR = stress reactivity (Arousal Predisposition Scale), AS = aberrant salience (Aberrant Salience Inventory), NB = negative beliefs about mistrust (Beliefs about Paranoia Scale - Negative Subscale), SA = social anxiety (Brief Fear of Negative Evaluation Scale), CF = cognitive fusion (Cognitive Fusion Questionnaire), ER = emotion regulation difficulties (Difficulties in Emotion Regulation Scale), H = hallucinations (Launey Slade Hallucinations Scale), O = ostracism (Ostracism Short Scale), PB = persecutory beliefs (Revised Green Paranoid Thoughts Scale), S = stress (Depression Anxiety Stress Scales - Stress Subscale), TA = threat anticipation (Negative Events Scale). Part A displays the network of the low persecutory beliefs sample, and Part B displays the network of the high persecutory beliefs sample.

Fig. 1 displays the network for A) low and B) high persecutory beliefs. The low persecutory beliefs network exhibited a mean edge weight of 0.08 and high density (42 of 55 possible undirected connections retained). The high persecutory beliefs network was comparably less dense, with 35 edges retained and a mean edge weight of 0.07. Regarding the explained variance (Q1), the predictability of low persecutory beliefs (*R*^*2*^ = 0.32) was higher than that of high persecutory beliefs (*R*^*2*^ = 0.17).

### 3.3 Direct associations with persecutory beliefs

Differences in the number and magnitude of direct edges involving persecutory beliefs (PB) emerged between the low and high persecutory beliefs networks (Q2). The edge weights for both networks are presented in Table 3. In the low persecutory beliefs network, three nodes had direct edges to persecutory beliefs: aberrant salience (AS), ostracism (O), and threat anticipation (TA). In the high persecutory beliefs network, five direct connections emerged between persecutory beliefs and negative beliefs about mistrust (NB), hallucinations (H), ostracism (O), stress (S), and threat anticipation (TA). Bootstrapped 95% confidence intervals were calculated to judge the stability of the edge weights (see Supplementary Fig. S2 for details). Although most edges were estimated reliably (i.e., confidence intervals did not encompass zero), interpreting the rank order of edge weights should be done cautiously due to considerable overlap among the confidence intervals.

**Table 3:**
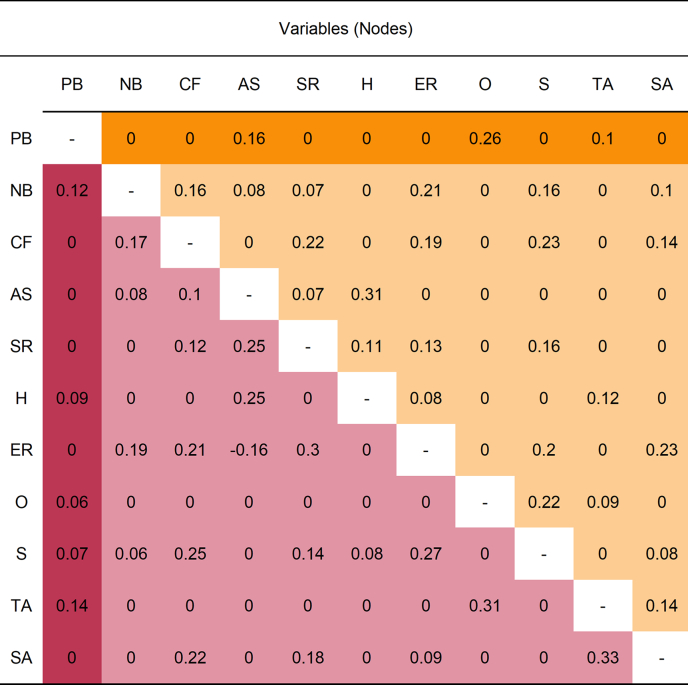
Edge weights (undirected regularised partial correlations) of the low and high persecutory beliefs network models.

*Note*. PB = persecutory beliefs (Revised Green Paranoid Thoughts Scale), NB = negative beliefs about mistrust (Beliefs about Paranoia Scale – Negative Subscale), CF = cognitive fusion (Cognitive Fusion Questionnaire), AS = aberrant salience (Aberrant Salience Inventory), SR = stress reactivity (Arousal Predisposition Scale), H = hallucinations (Launey Slade Hallucinations Scale), ER = emotion regulation difficulties (Difficulties in Emotion Regulation Scale), O = ostracism (Ostracism Short Scale), S = stress (Depression Anxiety Stress Scales - Stress Subscale), TA = threat anticipation (Negative Events Scale), SA = social anxiety (Brief Fear of Negative Evaluation Scale).

### 3.4 Centrality indices of persecutory beliefs

**Fig. 2:**
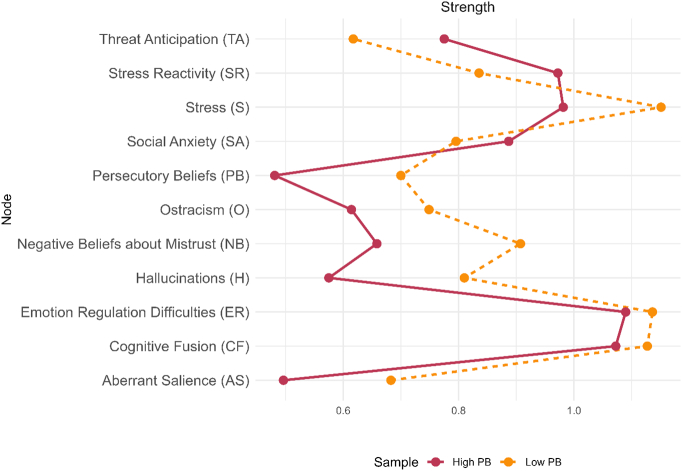
*Node strength of the nodes within the networks of individuals with low versus high persecutory beliefs*. *Note*. PB = persecutory beliefs. Node strength is the magnitude of all direct associations between a node and other nodes in the network.

To estimate the stability of the networks' centrality indices, case-dropping bootstrapping was performed (see Supplementary Material for further details). The *CS-coefficients* of *betweenness* (low persecutory beliefs: *CS*(cor = 0.7) = 0; high persecutory beliefs: *CS*(cor = 0.7) = 0.13) and *closeness* (low persecutory beliefs: *CS*(cor = 0.7) = 0.29; high persecutory beliefs: *CS*(cor = 0.7) = 0.36) were below the recommended cutoff of 0.5 (Epskamp et al., 2017). The *CS*-coefficient for *strength* was 0.52 for the low persecutory beliefs and 0.60 for the high persecutory beliefs network. This suggests that the rank order of the node strengths in both models can be cautiously interpreted, whereas the rank orders of betweenness and closeness are not reliably interpretable (Epskamp et al., 2017). Accordingly, only strength centrality was compared and is shown in Fig. 2. For completeness, an overview of the closeness and betweenness indices is provided in the Supplementary Material (Fig. S3). The strength of the low persecutory beliefs node (0.70) was larger than that of the high persecutory beliefs node (0.48), indicating that low persecutory beliefs were more strongly connected to other nodes in the network.

## 4 Discussion

This study utilised network analyses to explore differences in predicting low versus high levels of persecutory beliefs based on 55 theory-derived predictors of delusions (Denecke et al., 2025). This secondary analysis demonstrated that variance in low levels of persecutory beliefs was better accounted for by the selected predictors than variance in clinically high levels. Also, the strength of the persecutory beliefs node was higher in the low network than in the high network, indicating that more pronounced persecutory beliefs were less connected to the other variables. Furthermore, there were differences in associative patterns between the two subsamples: In individuals with low levels of persecutory beliefs, persecutory beliefs were directly associated with aberrant salience, threat anticipation, and ostracism. Aberrant salience and hallucinations were most strongly associated in the low subsample. In individuals with high persecutory beliefs, negative beliefs about paranoia, threat anticipation, hallucinations, ostracism, and stress were directly associated with persecutory beliefs. The strongest connections in this subsample emerged between social anxiety, threat anticipation, and ostracism.

The considerable difference in explained variance of paranoia scores in the low versus high persecutory beliefs samples suggests that predictors from existing aetiological models of delusions may be better suited to capture subclinical persecutory beliefs. The continuum view, as well as theoretical models of delusions, appear to implicitly assume linear relationships across the continuum, with higher predictor scores associated with higher persecutory beliefs in a similar manner across the continuum (e.g., Bebbington et al., 2013; Elahi et al., 2017; Freeman et al., 2005). Instead, we find a larger proportion of unexplained variance in persecutory beliefs above a clinical threshold. These findings do not support the idea that the predictors account for different levels of persecutory beliefs in a simple linear manner. If differences in associated factors exist across levels on the continuum, this challenges the frequent assumption that findings from subclinical samples can be directly generalised to clinical subgroups. While Elahi and colleagues (2017) proposed that the same dimensional structure applies across both clinical and subclinical groups, our data do not support this view and indicate that cross-sectional findings in subclinical samples cannot be directly extrapolated to clinical groups. This resonates with findings of Hajdúk and colleagues (Hajdúk et al., 2019), who reported differences in the mechanisms underlying interpersonal dysfunction comparing non-clinical populations and patients with schizophrenia spectrum disorders. Specifically, interpersonal difficulties were associated with negative beliefs about others in non-clinical populations, whereas negative experiences were related to interpersonal dysfunction in the clinical group. This suggests that different predictors and mechanisms may be implicated at different points along the continuum (Hajdúk et al., 2019). Further, and even more importantly, our findings point out the need to augment our understanding of the predictors of more severe persecutory beliefs.

Beyond differences in explained variance, the persecutory beliefs node also demonstrated higher centrality, specifically strength (i.e., the combined magnitude of its direct associations with other variables), within the low persecutory beliefs network, indicating that the node was more strongly connected to the predictors in the network of individuals with lower severity. This finding raises the possibility that once persecutory beliefs become more severe, they may become self-sustaining with diminished reliance on external predictors. Thus, while the predictors may be relevant to the clinical picture of individuals with persecutory beliefs, they may not directly influence persecutory beliefs. This interpretation is consistent with dynamic network approaches to psychopathology (Borsboom et al., 2019; Borsboom, 2017). In this dynamic approach, complex interactions among symptoms can give rise to self-sustaining feedback loops, which may render predictors differentially relevant for symptomatology across different stages of delusional aetiology.

Consistent with this dynamic view, the relative importance and connectivity of predictors varied across levels of persecutory beliefs within our networks, with some key differences warranting attention. Specifically, ostracism was particularly important at the lower end of the continuum, suggesting that negative social experiences of feeling excluded could be a starting point for persecutory beliefs to manifest. Once the persecutory beliefs reach a higher severity level, this dynamic might shift to an indirect relationship, in which beliefs are disconnected from real-life experiences but exacerbated and maintained by constant anticipation of threat. In a similar vein, our two networks indicated a shift in the relevance of perceptual predictors: Aberrant salience was more strongly associated with persecutory beliefs at the lower end of the distribution, suggesting that more vague perceptual anomalies might fuel early persecutory ideas. At the high end of the spectrum, hallucinatory experiences were more strongly associated with persecutory beliefs, suggesting a shift wherein more severe forms of perceptual aberrancies supersede more vague experiences the further individuals move up the continuum of persecutory beliefs. Interestingly, stress and negative beliefs about mistrust were directly associated with persecutory beliefs only in the high network. This could indicate a maintenance mechanism, whereby persecutory beliefs persist due to increased stress and worrying about the symptoms. Moreover, the networks revealed a negative association between aberrant salience and emotion regulation difficulties in the high persecutory beliefs network, whereas no association was observed in the low severity network. It seems, therefore, that cognitive–affective processes beyond those directly connected to persecutory beliefs may also change when paranoid beliefs become more severe. In light of these differences in associative patterns, rather than a linear relationship that would be inferred from the continuum view and theoretical accounts of delusions, there may be a more dynamic interplay among risk factors and predictors. Such a dynamic view underscores the need to diversify sample pools in future studies, including participants with varying severity levels across the continuum.

Several limitations of this study should be noted. Our data are cross-sectional and do not allow for directional or causal inferences. Interpretations thus remain speculative. A longitudinal follow-up study using temporal network models (e.g., using experience-sampling methods) would allow thorough analysis of the dynamic interplay of predictors across the continuum over time. The reported analyses are based on the same dataset as the previously reported machine learning results (Denecke et al., 2025). In addition, the analyses were not pre-registered and are exploratory. Thus, our findings require replication in a different sample. Furthermore, the limited explained variance could reflect a lack of predictors, particularly those explicitly associated with high-severity persecutory beliefs. It is possible that maintenance factors, such as safety behaviours, would at least partly fill the gap in explaining high persecutory beliefs, since recent studies have proposed their crucial role in maintaining, and perhaps even exacerbating, persecutory beliefs (Freeman and Loe, 2023). Future studies should incorporate maintenance mechanisms (Lincoln et al., 2026) to investigate whether they enhance explanatory power and provide a more comprehensive account of symptomatology at higher severity levels.

Despite these limitations, our findings indicate that predictors from aetiological models of delusions explain subclinical persecutory beliefs better than clinically high levels and with distinct association patterns. Tracking individuals over time and across the full spectrum of severity will be crucial to clarifying how different predictors gain or lose importance as persecutory beliefs intensify. Network analytic approaches that examine temporal dynamics may be well-suited to uncover the self-sustaining symptom interactions theorised in network models of psychopathology (Borsboom, 2017). For instance, temporal network models based on longitudinal (Berisha et al., 2025) and experience sampling data (Badal et al., 2021), as well as machine-learning-based causal discovery approaches (Stevenson et al., 2022; Sahin-Ilikoglu et al., 2024), represent promising methods for investigating potential directional relationships among predictors and symptoms. Applying such methods to the study of persecutory beliefs could advance our understanding of delusions and may ultimately inform personalised treatment efforts, for example, by identifying individual symptom networks and optimal points for intervention.

## CRediT authorship contribution statement

**S. Denecke:** Writing – review & editing, Writing – original draft, Methodology, Formal analysis, Conceptualization. **A. Bott:** Writing – review & editing, Methodology. **F. Strakeljahn:** Writing – review & editing, Methodology. **J. Kingston:** Writing – review & editing. **T.M. Lincoln:** Writing – review & editing, Conceptualization.

## Ethical statement

Ethical approval for the study procedure and data collection was obtained from the local ethics committee of the University of Hamburg (2022_029).

## Declaration of Generative AI and AI-assisted technologies in the writing process

During the preparation of this manuscript, AI language models (ChatGPT 5.2, OpenAI) were utilised for language editing. All content was reviewed, edited, and approved by the authors, who take full responsibility for the final version.

## Declaration of competing interest

The authors have no conflicts of interest to declare.

## Data Availability

The de-identified and aggregated data, as well as the R-code for the reported analyses, are available on the OSF (https://osf.io/7bzpa) and are subject to a CC BY-NC-SA 4.0 license.

## Appendix A Supplementary data

Supplementary material Image 1
