# Supplementary material for: Severity matters: Using network analysis to explain low and high levels of persecutory beliefs
Source: Schizophr Res Cogn. 2026 Mar 25;45:100435. doi: 10.1016/j.scog.2026.100435 (PMC13050109; doi:10.1016/j.scog.2026.100435)
Supplement: Supplementary file 1 — Supplementary material [file mmc1.docx]

**Severity Matters:**

**Using Network Analysis to Explain Low and High Levels of Persecutory Beliefs**

**- Supplementary Material -**

S. Denecke^a^*, A. Bott^a^, F. Strakeljahn^a^, J. Kingston^b^, & T. M. Lincoln^a^

^a^University of Hamburg, Clinical Psychology and Psychotherapy, Germany

^b^Royal Holloway University of London, Health and Wellbeing, United Kingdom

*Corresponding Author: Saskia Denecke, Von-Melle-Park 5, 20146 Hamburg, Germany, saskia.denecke@uni-hamburg.de

**Supplement 1**

**Table S1**

*Sample Characteristics by Low and High Severity Subgroup and in the Complete Sample.*

|  | **Mean (SD) or Frequency (%)** | | |
| --- | --- | --- | --- |
| **Variable** | **Low**^1^ | **High**^1^ | **Total Sample** |
| Gender |  |  |  |
| Male | 81 (48.2%) | 89 (53.0%) | 170 (50.6%) |
| Female | 83 (49.4%) | 78 (46.4%) | 161 (47.9%) |
| Non-binary | 3 (1.8%) | 1 (0.6%) | 4 (1.2%) |
| None apply | 1 (0.6%) | 0 (0.0%) | 1 (0.3%) |
| Age | 41.2 (14.0) | 37.8 (11.5) | 39.5 (12.9) |
| Education |  |  |  |
| Primary school | 0 (0.0%) | 2 (1.2%) | 2 (0.6%) |
| GCSE or equivalent | 26 (15.5%) | 21 (12.5%) | 47 (14.0%) |
| A-level or equivalent | 27 (16.1%) | 34 (20.2%) | 61 (18.2%) |
| Higher National Certificate | 21 (12.5%) | 17 (10.1%) | 38 (11.3%) |
| Bachelor's degree | 65 (38.7%) | 69 (41.1%) | 134 (39.9%) |
| Master's degree | 25 (14.9%) | 20 (11.9%) | 45 (13.4%) |
| PhD | 4 (2.4%) | 5 (3.0%) | 9 (2.7%) |
| Diagnosis of a Psychotic Disorder | 0 (0.0%) | 2 (1.2%) | 2 (0.6%) |
| Diagnosis of any other Mental Disorder | 48 (28.6%) | 62 (36.9%) | 110 (32.7%) |
| Receiving Mental Health Treatment | 24 (14.3%) | 42 (25.0%) | 66 (19.6%) |
| Ethnicity |  |  |  |
| Black | 2 (1.2%) | 4 (2.4%) | 6 (1.8%) |
| Asian | 7 (4.2%) | 11 (6.5%) | 18 (5.4%) |
| White | 154 (91.7%) | 139 (82.7%) | 293 (87.2%) |
| Mixed | 3 (1.8%) | 10 (6.0%) | 13 (3.9%) |
| Other | 1 (0.6%) | 2 (1.2%) | 3 (0.9%) |
| Unknown | 1 (0.6%) | 2 (1.2%) | 3 (0.9%) |
| Marital Status |  |  |  |
| Married | 52 (31.0%) | 48 (28.6%) | 100 (29.8%) |
| Divorced/Separated | 12 (7.1%) | 12 (7.1%) | 24 (7.1%) |
| In a relationship | 54 (32.1%) | 48 (28.6%) | 102 (30.4%) |
| Single | 48 (28.6%) | 59 (35.1%) | 107 (31.8%) |
| Unknown | 2 (1.2%) | 1 (0.6%) | 3 (0.9%) |
| ^1^n = 84, N = 336 | | |  |

**Figure S1**

*Absolute Correlations between Predictors for the Complete Sample.*


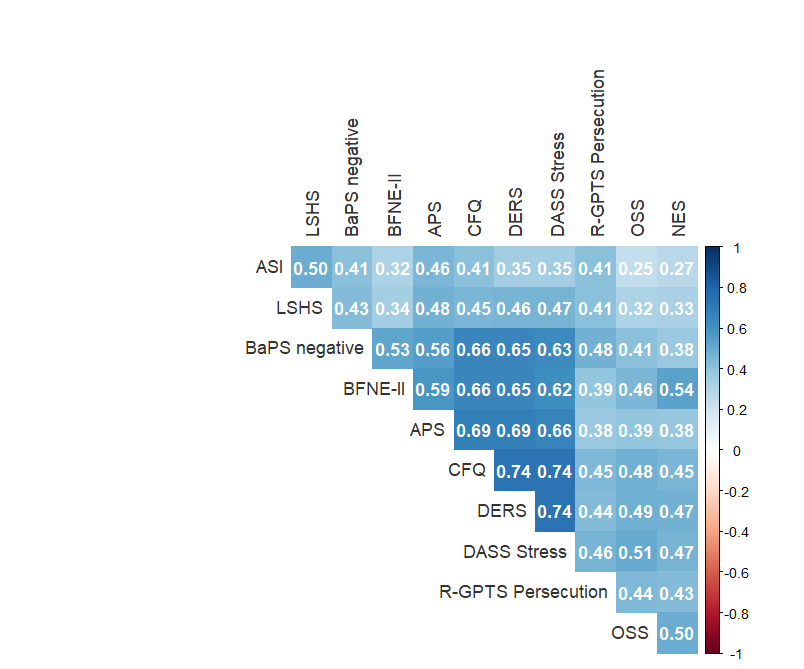


*Note*. ASI = aberrant salience (Aberrant Salience Inventory), CFQ = cognitive fusion (Cognitive Fusion Questionnaire), DERS = emotion regulation difficulties (Difficulties in Emotion Regulation Scale), LSHS = hallucinations (Launey Slade Hallucinations Scale), BaPS negative = negative beliefs about mistrust (Beliefs about Paranoia Scale - Negative Subscale), OSS = ostracism (Ostracism Short Scale), R-GPTS Persecution = persecutory beliefs (Revised Green Paranoid Thoughts Scale), DASS Stress = stress (Depression Anxiety Stress Scales - Stress Subscale), BFNE-II = social anxiety (Brief Fear of Negative Evaluation Scale), APS = Stress Reactivity (Arousal Predisposition Scale), NES = threat anticipation (Negative Events Scale)

**Figure S2**

*Bootstrapped Confidence Intervals (95%) of the Edge Weights for the A) Low and B) High Persecutory Beliefs Networks.*

| **A** | **B** |
| --- | --- |
| **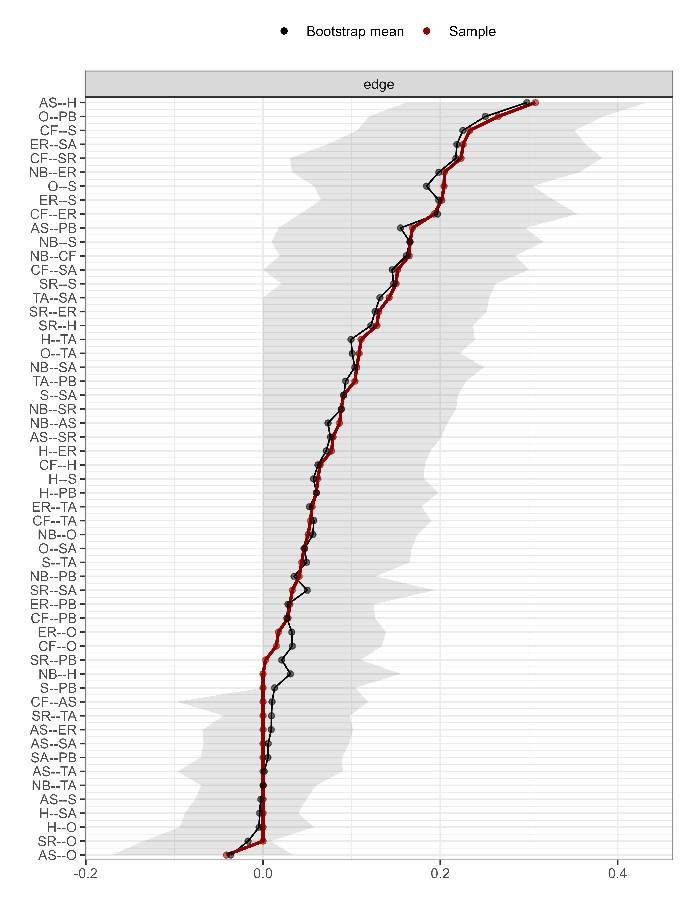** | 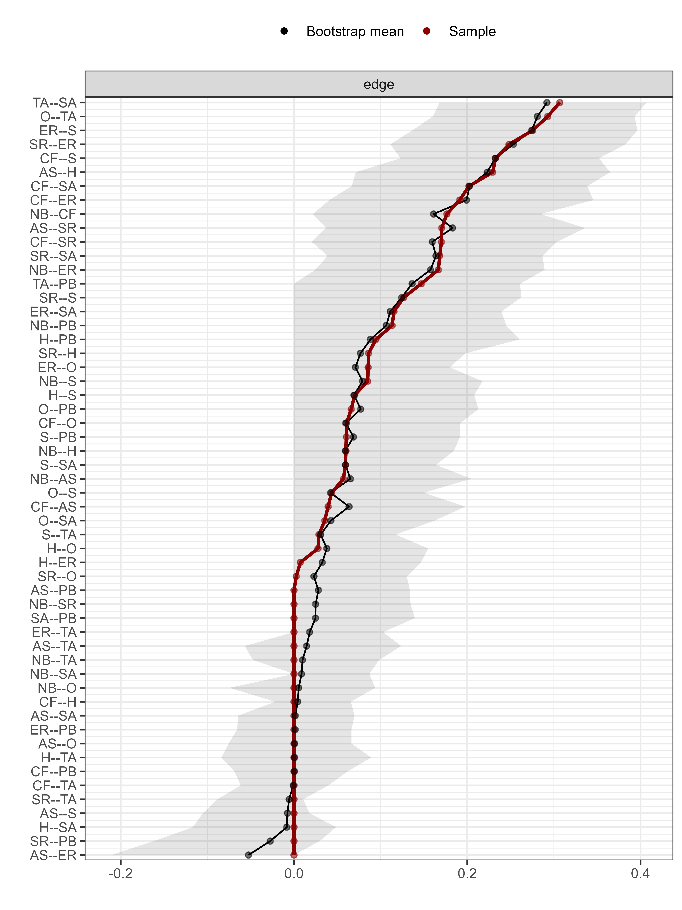 |

*Note*. Bootstrapping based on 1000 samples. AS = aberrant salience (Aberrant Salience Inventory), CF = cognitive fusion (Cognitive Fusion Questionnaire), ER = emotion regulation difficulties (Difficulties in Emotion Regulation Scale), H = hallucinations (Launey Slade Hallucinations Scale), NB = negative beliefs about mistrust (Beliefs about Paranoia Scale - Negative Subscale), O = ostracism (Ostracism Short Scale), PB = persecutory beliefs (Revised Green Paranoid Thoughts Scale), S = stress (Depression Anxiety Stress Scales - Stress Subscale), SA = social anxiety (Brief Fear of Negative Evaluation Scale), SR = Stress Reactivity (Arousal Predisposition Scale), TA = threat anticipation (Negative Events Scale).

**Figure S3**

*Centrality Indices of the Networks of Individuals with Low and High Persecutory Beliefs.*


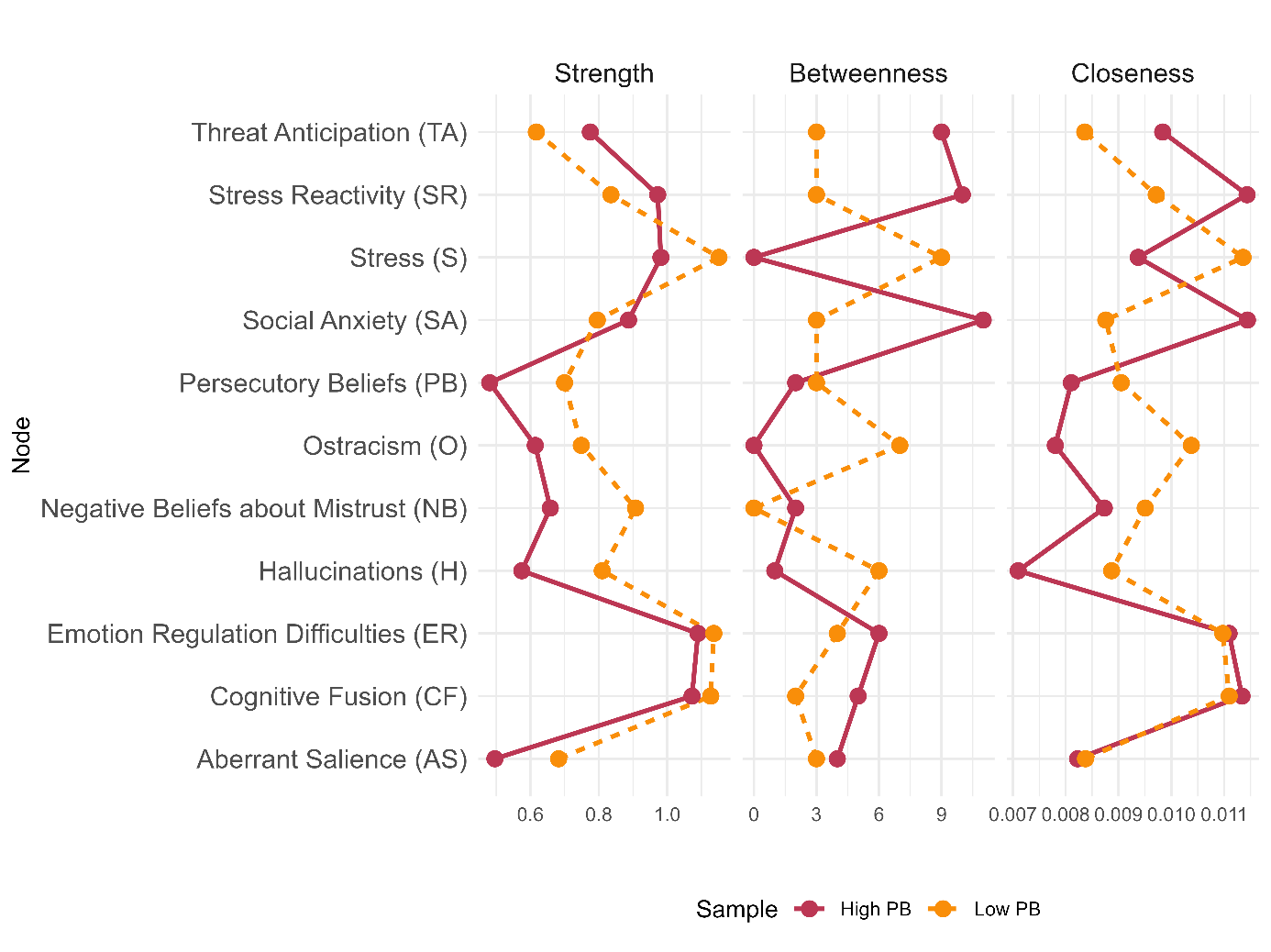


*Note*. PB = Persecutory Beliefs.

**Figure S4**

*Case-dropping Bootstrapped Centrality Indices for the A) Low and B) High Persecutory Beliefs Networks.*

**A**


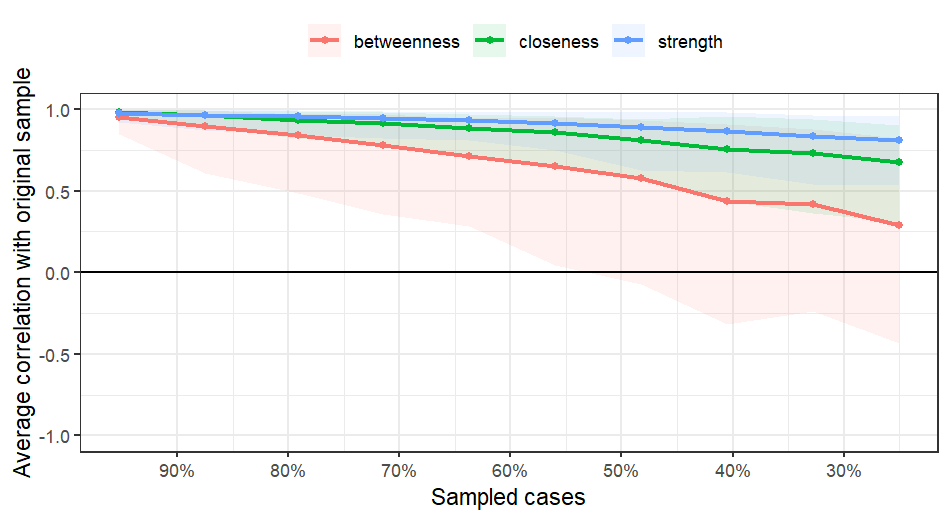


**B**


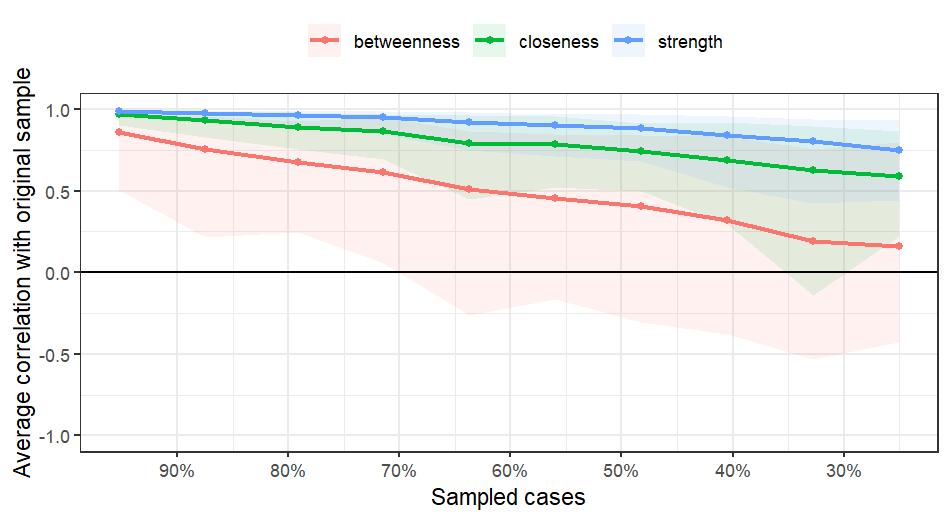


*Note*. Bootstrapping based on 1000 samples.
